# Two novel DNA motifs are essential for *BACE1* gene transcription

**DOI:** 10.1038/srep06864

**Published:** 2014-10-31

**Authors:** Yan Xiang, Shasha Meng, Jinfeng Wang, Songyang Li, Jingru Liu, Hongmei Li, Tingyu Li, Weihong Song, Weihui Zhou

**Affiliations:** 1 Ministry of Education Key Laboratory of Child Development and Disorders; Chongqing Key Laboratory of Translational Medical Research in Cognitive Development and Learning and Memory Disorders, Children's Hospital of Chongqing Medical University, 136 ZhongshanEr Lu, Yuzhong District, Chongqing 400014, China; 2 Townsend Family Laboratories, Department of Psychiatry, Brain Research Center, University of British Columbia, Vancouver, British Columbia, V6T1Z3, Canada

## Abstract

*BACE1* gene encodes for β-Site amyloid β precursor protein (APP)-cleaving enzyme1, which is required for generating amyloid β protein(Aβ). Deposition of Aβ in brain plays an essential role in Alzheimer's Disease (AD) pathogenesis. *BACE1* gene has a tissue-specific expression pattern and its expression is tightly regulated at transcriptional level. Core promoter is a minimal DNA sequence to direct transcription initiation and serves as a converging platform for the vast network of regulatory events. Here we identified the core promoter of human *BACE1* gene, which is a 71 nucleotides region absent of typical known core promoter elements and is sufficient to initiate a basal transcription. Two novel DNA motifs, designated TCE1 and TCE2, were found to be involved in activating the transcription of human *BACE1* gene in a synergistic way. Two single nucleotide mutations in these motifs completely abolished the promoter activity. In conclusion, our studies have demonstrated that novel DNA motif TCE1 and TCE2 in human *BACE1* gene promoter are two essential *cis*-acting elements for *BACE1* gene transcription. Studies on how these two motifs being regulated by different stimuli could provide insights into the molecular mechanisms underlying AD pathogenesis and pharmaceutical potentials of targeting these motifs for AD treatment.

Amyloid β protein (Aβ) is the major component of amyloid neuritic plaques in the brain of AD and its deposition in the brain plays an essential role in AD pathogenesis^1^. Aβ is generated from APP by β- and γ-secretase cleavages^2^. *BACE1* gene encodes for β-Site APP cleaving enzyme1 (BACE1, also known as Asp2 or memapsin2), a type 1 membrane-associated aspartyl protease of 501 amino acids^3^,^4^,^5^,^6^. BACE1 is a β-secretase *in vivo*^2^,^7^,^8^,^9^,^10^,^11^. BACE1 cleaves APP at two β-secretase sites, at Asp1 site to produce CTFβ C99 in amyloidogenic pathway, or at Glu11 site to produce CTFβ C89 in non-amyloidogenic pathway^12^. CTFβ C99 is the substrate of presenilin-dependent γ-secretase complex to produce Aβ, thus cleavage of APP by BACE1 at Asp1 site is essential in the amyloidogenic pathway. The β-cleavage is considered a rate limiting step for Aβ production. Degradation of BACE1 is mediated by the ubiquitin proteasome pathway^13^,^14^,^15^. A slight increase of BACE1 expression is sufficient to promote C99 and Aβ production under pathological conditions^16^. Reduction of Aβ by inhibition of BACE1 activity is considered to have major pharmaceutical potential for AD therapy^2^,^17^,^18^.

The *BACE1* gene has a tissue and stimuli-specific expression pattern^2^,^16^. *BACE1* mRNA has been found mainly in neuronal cells and in the pancreas^3^,^4^. *BACE1* expression is tightly regulated at the level of transcription^19^. The upstream sequence of the *BACE1* gene contains many putative transcription factor binding sites^19^,^20^,^21^. Several transcription factors (TFs) have been experimentally confirmed to bind to the regulatory region of *BACE1* gene and to positively regulate its expression. Transcription factor specificity protein 1 (Sp1) was the first one discovered to play an important role in regulating human *BACE1* expression^19^. Hypoxia inducible factor 1(HIF-1) was found to bind to a hypoxia responsive element (HRE) on *BACE1* promoter and mediated the increases of *BACE1* transcription induced by hypoxia^22^,^23^,^24^,^25^. Signal transducer and activator of transcription 1 (STAT1) is responsible for *BACE1* constitutively expression in neurons and inducible expression by interferon γ (IFNγ) in astrocytes^26^,^27^. Transcriptional factor Yin Yang 1(YY1) and nuclear factor of activated T-cells 1 (NFAT1) were also reported to up-regulating *BACE1* transcription^28^,^29^,^30^. There are also many TFs and their binding sites down-regulating *BACE1* gene transcription. Two GATA-1 sites located at -106 to -97 and -209 to -200 from authentic ATG codon of rat *BACE1* were found to act as suppressors of *BACE1* transcription^21^. The peroxisome proliferator-activated receptor-γ (PPARγ) can bind to PPARγ responsive element(PPRE) of rat *BACE1* promoter and suppress *BACE1* transcription, and decreasing PPARγ level by proinflammatory cytokines is responsible for activation of *BACE1* promoter^31^,^32^. Functional binding sites of Nuclear factor κB (NFκB) were identified in *BACE1* promoter region^21^,^33^,^34^. NFκB was reported to differentially regulate *BACE1* gene transcription, which depends on cell-type or cell-condition^23^,^35^,^36^. Under physical condition or in neuronal and non-activated astrocytic cells activated NFκB inhibits *BACE1* gene expression, while it activates *BACE1* in higher Aβ concentrations or in activated astrocytes. The majority of cis-acting elements of BACE1 gene promoter are located far from transcriptional start site (TSS). There is little research on the function and structure of the proximal region of *BACE1* promoter or core promoter near TSS.

Core promoter is a minimal DNA sequence to direct transcription initiation and serves as a converging platform for the vast network of regulatory events by interaction of transcription factors and proximal or distal regulatory DNA motifs in the promoter^37^,^38^. Based on the distribution of TSS, core promoter was divided into two classes as focused (sharp) and dispersed (broad) promoter^37^,^39^. In general, a focused promoter is associated with genes containing typical core promoter elements (TATA, Inr, DPE or MTE), lacks positioned nucleosomes in the promoter region and has a labile transcriptional state and a low GC content; whereas a dispersed promoter correlates with housekeeping genes with positioned nucleosomes in the promoter region, and it does not have known core promoter elements, and has a stable transcriptional state and a high GC content^37^,^38^. Analysis of focused core promoter in RNA polymerase II (RNAPII)-transcribed genes suggests that a core promoter typically spans from around nucleotide −40 to +40 relative to transcription start site and consists of functional sub-regions termed core promoter elements such as Inr, TATA box, BRE,DPE,MTE, XCPE1. Recent evidence indicates that different core promoter type can interact with different transcription components and such diversity plays an important role in driving cell-specific transcription and regulatory events^40^. *BACE1* gene promoter lacks the typical CAAT and TATA boxes within 2 kb upstream region of the TSS^19^. In-depth analysis of structure and function of *BACE1* gene core promoter will greatly advance our understanding the transcriptional mechanisms of TATA-less core promoter and may benefit for developing new strategies in inhibiting *BACE1* expression in prevention and treatment of Alzheimer's disease.

In this study, we defined the boundary of the proximal region of human *BACE1* promoter, which is a 104 base pairs in length and sufficient for high levels of expression in cells. We identified the core promoter region of 71 nucleotides from −550 to −480 bp relative to ATG of the *BACE1* gene and this core promoter region is able to initiate a basal transcription of downstream gene. *BACE1* gene core promoter lacks known core promoter elements. Two novel DNA motifs, designated as TCE1 and TCE2, were found to be involved in activating the transcription of *BACE1* gene in a synergistic way. Reducing the spacing between TCE1 and TCE2 decrease the promoter activity and two single nucleotide mutations in these motifs are able to completely abolish promoter activity of *BACE1* gene. Collectively, our studies have demonstrated that novel DNA motif TCE1 and TCE2 identified in human *BACE1* gene promoter are essential for controlling transcription of *BACE1* gene.

## Results

### Determination of the boundary of BACE1 minimal and core promoter

Our previous study demonstrated that the region from −1311 to −400 bp upstream of the translation start codon (as +1) of the human *BACE1* gene has promoter activity^19^. In order to identify core promoter, first we constructed luciferase reporter plasmid p3BU-1273/-400 containing sequence −1273 to −400 bp region upstream of translating start site of *BACE1* gene inserted in pGL3-Basic vector. DNA sequencing confirmed that the sequence of inserted *BACE1* –1273 to -400 in p3BU-1273/-400 is identical to 117187784-117186911 of chromosome 11 (GRCh37/hg19), except for one SNP rs536817 (chr11:117187441), which is G in our sequence. The pGL3-Basic vector lacks eukaryotic promoter and enhancer sequences upstream of a reporter luciferase gene. Luciferase activity in cells transfected with reporter plasmid depends on the expression of luciferase gene, and the expression level depends on insertion and proper orientation of a functional promoter upstream from the reporter luciferase gene.

**Figure 1:**
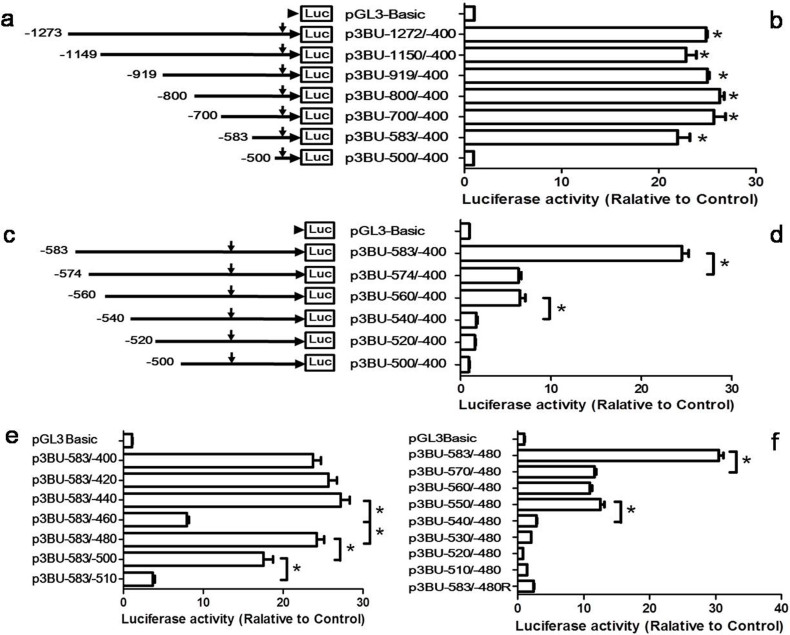
Functional end deletion analysis of human *BACE1* gene promoter. (a)(c) Schematic diagram of plasmid constructs. The horizontal line indicates the *BACE1* promoter region; horizontal arrow indicates transcriptional direction and the downward arrow indicates the *BACE1* transcriptional start site. The box LUC represents the coding sequence of the luciferase reporter gene. The numbers indicate the endpoints of each construct, with +1 as the adenine of translational start codon of the *BACE1* gene. (b)(d)(e)(f) HEK293 cells were transiently co-transfected with the deletion plasmids and p3PRluc. Firefly luciferase activity was measured 24 h after transfection, and Renilla luciferase activity was used to normalize for transfection efficiency. Data are shown as folds over the samples transfected with empty control pGL3basic plasmid. The values represent means standard error of the mean (n = 3–4). * P< 0.05 by analysis of variance (ANOVA) with the post-hoc Newmann-Keuls test.

To identify the boundary of minimal 5′ flanking region required for *BACE1* promoter activity, several deletion plasmids were generated and transfected into HEK293 cell. Luciferase activity measured from cells transfected with the original pGL3-Basic vector served as control. The results of luciferase assay indicated that p3BU-1273/-400, p3BU-1149/-400, p3BU-919/-400, p3BU-800/-400, p3BU-700/-400, p3BU-583/-400 have similar promoter activity and are significantly higher than pGL3-Basic control (Fig. 1a, b). Further deletion of 83 bp (p3BU-500/-400) almost abolished luciferase activity. This indicates that the 184 bp fragment (-583/-400) contains the functional promoter region and the 83 bp long region between -583 to -501 contains important *cis*-acting elements required for human *BACE1* gene transcription. To narrow down this important region, more deletion plasmids were constructed and luciferase assay (Fig. 1c, d) showed that further deleting 10 bp from 5′end of -583/-400 drastically reduced luciferase activity, but is still higher than negative control. Additional deletion of 14 bp (p3BU-560/-400) displayed similar luciferase activity as p3BU-574/-400. However, continuous deletion of 20 bp (p3BU-540/-400) caused significant decrease of luciferase activity, which is close to the levels of pGL3-Basic control. The results indicate that region -583 to -574 and -560 to -540 have important and functional elements required for BACE1 promoter activity.

Since the region -583 to -400 has significant promoter activity and further deletion from 5′ end causes drastically decrease of activity, p3BU-583/-400 was chosen as a template to investigate the effect of 3′ end on promoter activity. A series of 3′end deletion plasmids were constructed and transfected into HEK293 cell. Luciferase assay showed that deleting two 20 bp from 3′end of -583/-400 (resulting in p3BU-583/-420 and p3BU-583/-440, respectively) had no significant effects on luciferase activity (Fig. 1e). Further deletion of 20 bp (p3BU-583/-460) caused significant decrease of luciferase activity compared to p3BU-583/-440. However, deleting additional 20 bp from 3′end of -583/-460 (p3BU-583/-480) surprisingly recovered luciferase activity to almost same as p3BU-583/-400. Further deleting 20 bp from 3′end of -583/-480 significantly reduced luciferase activity and continuous deletion of another 10 bp (p3BU-583/-510) caused further decrease of luciferase activity and is closed to the levels of pGL3-Basic control (Fig. 1e). It suggests that -480 is the 3′boundary of region required for *BACE1* minimal promoter activity and the region of -510 to -480 is essential for *BACE1* promoter activity.

To validate whether the position of nucleotide -480 is the 3′ boundary of region required for *BACE1* minimal promoter activity in the different context of 5′ end, more 5′ end deletion plasmids were generated and transfected into HEK293 cell. Luciferase activity showed that a series of plasmids ended with -480 as 3′end have similar pattern of luciferase activity as a series of plasmids 3′ ended with -400 (Fig. 1f). Deleting 14 bp from 5′end of *BACE1* fragment -583 to -480 drastically reduced luciferase activity, further deleting two 10 bp region (p3BU-560/-480 and p3BU-550/-480) showed similar activity as p3BU-570/-480 and continuing to delete 10 bp from 5′end of -550 to -480 caused further significant decrease of luciferase activity in p3BU-540/-480. It suggested that 71 bp region of -550 to -480 still has significant promoter activity. Inserting the 104 bp fragment of -583 to -480 bp in reverse direction in plasmid p3BU-583/-480R abolished luciferase activity (Fig. 1f). Taken together, our data demonstrated that this 104 nucleotides region from -583 to -480 bp upstream of the translational start site is sufficient for high level expression of *BACE1* gene and is the *BACE1* minimal promoter, and furthermore, the 71 nucleotides region of -550/-480 bp functions as *BACE1* core promoter, which is responsible for determining the basal transcription of *BACE1* gene.

### Identification of the cis-acting regions

**Figure 2:**
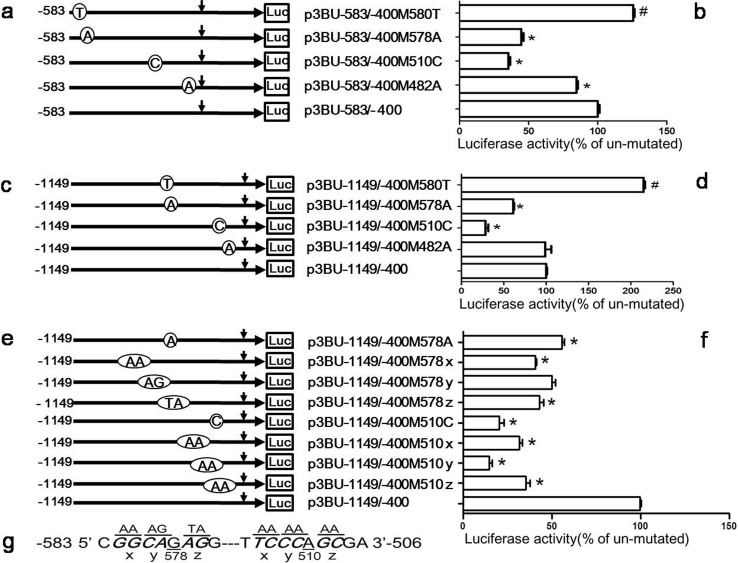
Identification of the cis-acting regions. (a)(c)(e) Schematic diagram of plasmid constructs containing the *BACE1* gene promoter with random single nucleotide mutation or cluster mutation with the plasmid p3BU-583/-400 or p3BU-1149/-400 as template. The horizontal line indicates the *BACE1* promoter region; horizontal arrow indicates transcriptional direction and the downward arrow indicates the *BACE1* transcriptional start site. Open circle with letter in it indicates mutation. The box LUC represents the coding sequence of the luciferase reporter gene. The numbers indicate the endpoints of each construct, with +1 as the adenine of the physiological translational start codon of the *BACE1* gene. (b) (d) (f) HEK293 cells were transiently transfected reporter plasmids. Firefly luciferase activity was measured 24 h after transfection, and Renilla luciferase activity was used to normalize for transfection efficiency. Data are shown as percentage of control samples transfected with wild-type plasmid. The values represent means standard error of the mean (n = 3–6). # or * P< 0.05 by analysis of variance (ANOVA) with the posthocNewmann-Keuls test when comparing with control. (g) Schematic diagram of nucleotide cluster mutation around position -578 and -510.

Our study suggests that there are a few sub-regions in *BACE1* minimal promoter sequence important for *BACE1* gene transcription, especially the region -583 to -574 and -510 to -480. To further investigate whether these regions are functional, reporter plasmids with random single nucleotide mutation were constructed with plasmid p3BU-583/-400 as template and the mutant plasmids were transfected into HEK293 cell. Luciferase assay showed that the single nucleotide mutation from C to T at -580 (-580C/T) had no negative effects, but mutation -578G/A, -510A/C or -482T/A negatively affected luciferase activity of plasmid p3BU-583/-400 (Fig. 2a and b).

Since position -580 and -578 are very close to the vector sequence in plasmid p3BU-583/-400M580T and p3BU-583/-400M578A, in order to exclude the possible effects of flanking vector sequence on luciferase activity of inserted fragment and verify the effects of those mutation, plasmid p3BU-1149/-400 carrying the mutations with extended 5′ end were generated (Fig. 2c). Luciferase assay showed that mutations 578G/A, 510A/C still significantly reduced the luciferase activity of wildtype plasmid p3BU-1149/-400 (Fig. 2d). Next, the effects of surrounding nucleotides of nucleotide -578G and -510A were investigated to determine if there are *cis*-acting regions in the vicinity of these two nucleotides. Several nucleotide cluster mutation plasmids were constructed as shown in Fig. 2g and 2e. Luciferase assay demonstrated that five cluster mutations near nucleotide -578G and -510A significantly reduced the luciferase activity of wildtype plasmid p3BU-1149/-400 (Fig. 2f) and suggested that there are functional *cis*-acting regions near -578G or -510A, respectively.

### The *cis*-acting regions have a synergistic effect on activating *BACE1* transcription

**Figure 3:**
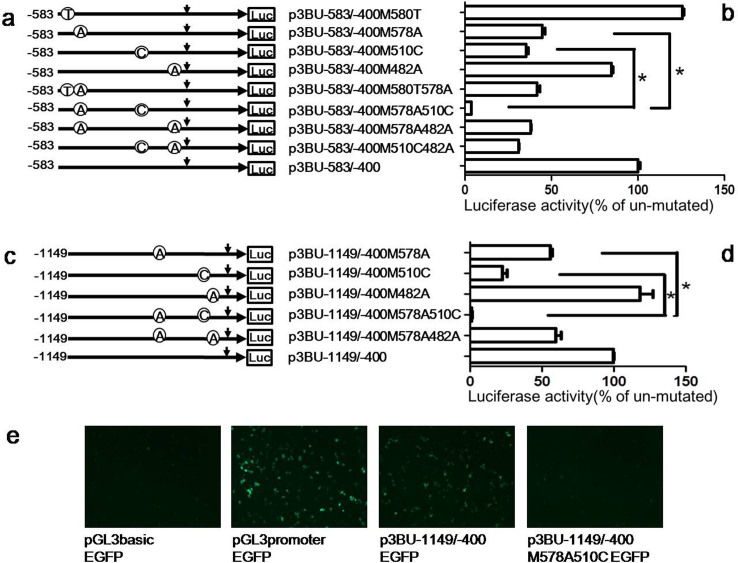
The cis-acting regions have a synergistic effect on activating *BACE1* transcription. (a)(c) Schematic diagram of plasmid constructs containing the *BACE1* gene promoter with double nucleotide mutations with the plasmid p3BU-583/-400 or p3BU-1149/-400 as template. The horizontal line indicates the *BACE1* promoter region; horizontal arrow indicates transcriptional direction and the downward arrow indicates the *BACE1* transcriptional start site. Open circle with letter in it indicates mutation. The box LUC represents the coding sequence of the luciferase reporter gene. The numbers indicate the endpoints of each construct, with +1 as the adenine of the physiological translational start codon of the *BACE1* gene. (b) (d) HEK293 cells were transiently transfected reporter plasmids. Firefly luciferase activity was measured 24 h after transfection, and Renilla luciferase activity was used to normalize for transfection efficiency. Data are shown as percentage of control samples transfected with wild-type plasmid. The values represent means standard error of the mean (n = 3–6). # or * P< 0.05 by analysis of variance (ANOVA) with the posthocNewmann-Keuls test when comparing with control. (e) HEK293 cells were transiently transfected EGFP reporter plasmids. Fluorescence was observed 24 h after transfection.

To investigate whether the afore-mentioned different regions have a synergistic effect on *BACE1* promoter activity, reporter plasmids containing single nucleotide mutations in both regions were constructed with plasmid p3BU-583/-400 as template and transfected into HEK293 cell. Compared with single nucleotide mutation of 578G/A (p3BU-583/-400M578A) or 580C/T (p3BU-583/-400M580T), double nucleotides mutation of 578G/A and 580C/T (p3BU-583/-400M580T578A) showed similar luciferase activity as single nucleotide mutation of 578G/A, suggesting that mutation of 580C/T has no additional effects on luciferase activity of plasmid p3BU-583/-400M578A (Fig. 3a,b). However, luciferase assay showed that double mutations 578G/A and 510A/C (p3BU-583/-400M578A510C) resulted in the strongest negative effect on luciferase activity and the luciferase activity of the double mutant plasmid was much lower than that of each single nucleotide mutation of 578G/A or 510A/C (Fig. 3a,b). Compared with single nucleotide mutation of 578G/A (p3BU-583/-400M578A) or 482T/A (p3BU-583/-400M482A), double nucleotides mutation of 578G/A and 482T/A (p3BU-583/-400M578A482A) showed similar luciferase activity as single nucleotide mutation of 578G/A, suggesting that mutation of 482T/A had no effects on promoter activity. The luciferase assay of the double mutant 510A/C and 482T/A (p3BU-583/-400M510C482A) also suggest that the mutation 482T/A had no significant effects on *BACE1* gene transcription (Fig. 3a, b).

Next, we examined the possible effects of flanking vector sequence on luciferase activity. Compared with single nucleotide mutation of 578G/A (p3BU-1149/-400M578A) or 510A/C (p3BU-1149/-400M510C), double mutant 578G/A and 510A/C (p3BU-1149/-400M578A510C) drastically reduced luciferase activity of wildtype plasmid p3BU-1149/-400 and had much lower luciferase activity than each single mutant 578G/A or 510A/C (Fig. 3c, d). To further verify the significant effects of the double mutation of 578G/A and 510A/C on *BACE1* gene transcription, four plasmids with green fluorescent protein (EGFP) as a reporter gene were constructed by replacing the luciferase reporter gene with EGFP gene in plasmids pGL3-Basic, pGL3-Promoter, p3BU-1149/-400 and p3BU-1149/-400M578A510C. Empty control plasmid pGL3basic-EGFP didn't show florescence 24 hours after transfection into HEK293 cell, positive plasmid pGL3promoter-EGFP, containing a strong SV-40 promoter, showed very strong florescence. Plasmid p3BU-1149/-400EGFP displayed significant brighter florescence than pGL3basicEGFP. As expected, plasmid p3BU-1149/-400M578A510CEGFP displayed much weaker florescence than p3BU-1149/-400EGFP (Fig. 3e). These results confirmed that combining two single nucleotide mutations of 578A and 510C had a synergistic effect on *BACE1* promoter activity. The data suggest that the two *cis*-acting regions near nucleotides -578G and -510A play a pivotal role and function cooperatively in determining *BACE1* gene transcription.

### Identification of the nucleotide composition for the two cis-acting elements

**Figure 4:**
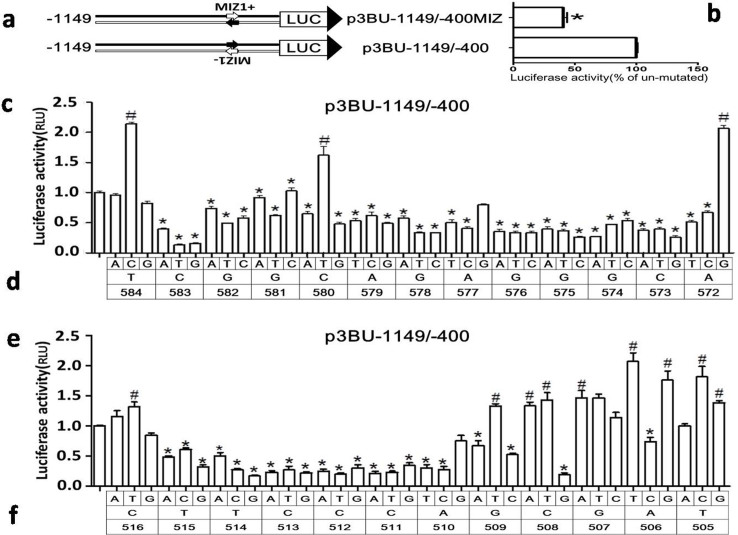
Identification of two transcription critical elements for *BACE1* gene. (a) Schematic diagram of plasmid constructs. The two horizontal lines indicates two DNA strands of the *BACE1* promoter region; inverted letter of MIZ1- indicate its original orientation at minus strand; small arrow indicates MIZ1 motif. The box LUC with large arrowhead represents the coding sequence of the luciferase reporter gene and transcriptional direction. The numbers indicate the endpoints of each construct, with +1 as the adenine of the physiological translational start codon of the *BACE1* gene. (b) (c) (e) HEK293 cells were transiently transfected reporter plasmids. Firefly luciferase activity was measured 24 h after transfection, and Renilla luciferase activity was used to normalize for transfection efficiency. Data are shown as percentage of control samples transfected with wild-type plasmid. The values represent means standard error of the mean (n = 3–6). # or * P< 0.05 by analysis of variance (ANOVA) with the post-hoc Newmann-Keuls test when comparing with control. (d) (f) Schematic diagram of plasmid constructs containing each nucleotide substitution in plasmid p3BU-1149/-400. Top row indicate nucleotide substitution; middle row indicate wild-type nucleotide and bottom row indicate the nucleotide position.

In order to identify possible proteins binding to surrounding sequences of nucleotide -578G or -510A, a 21-nucleotide sequence corresponding to 10 nucleotides flanking each side of -578G or -510A was used for transcriptional factor analysis using MatInspector^41^. Only one protein Myc-interacting Zn finger protein 1 (MIZ1) was found to match the sequence in *BACE1*-588/-568 (CACCTCGGCAGAGGGCATCCC). The MIZ1-binding sequence identified by MatInspector is gatgcCCTCtg (capital letter represents core sequence), which is located in antisense strand of *BACE1*-580 to -570 (CAGAGGGCATC). Since the candidate binding site of MIZ1 is located in antisense strand of *BACE1* gene, a reporter plasmid (p3BU-1149/-400MIZ) with candidate binding site of MIZ1 at sense strand was constructed and transfected into HEK cells. The luciferase activity of p3BU-1149/-400MIZ was significantly lower than wildtype p3BU-1149/-400(Fig. 4a, b), suggesting that candidate MIZ1 binding site identified by MatInspector may not be functional. Searching transcriptional factors binding to *BACE1* gene promoter sequence of -520/-500 (TACACTTCCCAGCGATCCCAG) with program MatInspector resulted in no hit.

Since there is no known transcriptional factor bound to these two important regions, next we defined the boundaries of both regions and investigated the effects of nucleotide substitution on promoter activity. We first generated p3BU-1149/-400 mutant plasmids carrying mutations surrounding -578G or -510A, and each nucleotide was replaced with three other nucleotides, respectively. The luciferase assay results showed that the majority of nucleotide replacements in region between -583 and -573 had negative effects on promoter activity (Fig. 4c), indicating that the region between -583 and -573 contains a positive-regulatory *cis*-acting element and the sequence of CGGCAGAGGGC was named as *BACE1* Transcription Critical Element 1 (TCE1), which may bind to unknown transcriptional factor. The nucleotide replacement at -584T with A or G has the similar luciferase activity as wildtype and the nucleotide replacement at -584T with C and at -572A with G even increased the promoter activity. The nucleotide replacement at -580C with T also significantly increase the promoter activity, suggesting that the wildtype sequence of TCE1 may not be the ideal sequence for binding with a transcriptional factor.

For the functional region surrounding -510A, the boundary seems to be -515 and -509, since almost every replacement between -515 and -509 significantly reduce the promoter activity (Fig. 4d). However, the replacement at -516C with three other nucleotides and the majority of nucleotide replacement between -508 and -505 had no negative effects. The region between -515 and -509 contains a positive-regulatory *cis*-acting element and the sequence of TTCCCAG was named as *BACE1* Transcription Critical Element 2 (TCE2). Comparing the effects between TCE1 and TCE2 on *BACE1* promoter activity, many nucleotide replacements in TCE2 caused 70% reduction and nucleotide replacements in TCE1 only caused 40-50% reduction, suggesting that TCE2 has stronger effects than TCE1 on *BACE1* transcription.

### TCE1 is highly conserved and serves as a promoter proximal element

**Figure 5:**
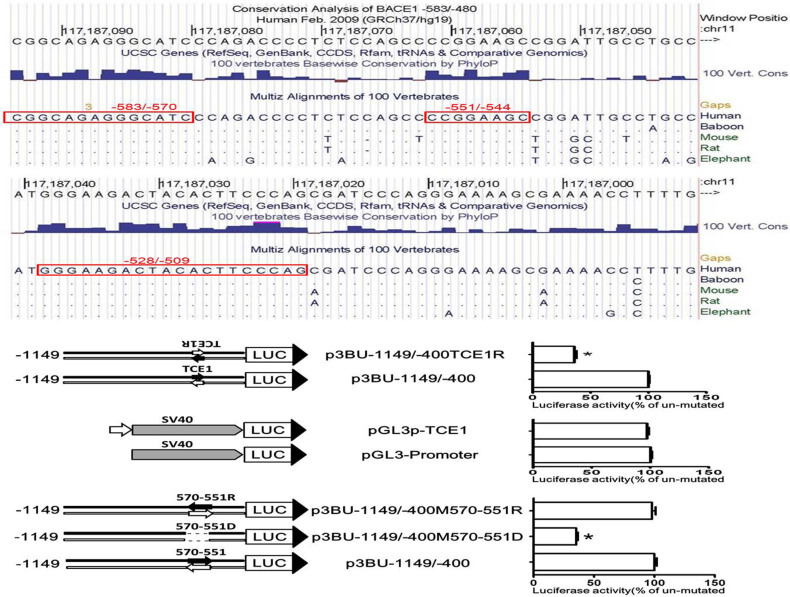
TCE1 is highly conserved and served as a promoter proximal element. (a) Conservation analysis of the *BACE1*-583/-480 among the difference species. Red box indicates high conserved sequences. (b) (d) (f) Schematic diagram of plasmid constructs. The box LUC with large arrowhead represents the coding sequence of the luciferase reporter gene and transcriptional direction; the horizontal line indicates upstream region of luciferase gene in reporter plasmids; short dashed line indicate deleted region; small arrow indicates interested DNA motif. (c) (e) (g) HEK293 cells were transiently transfected reporter plasmids. Firefly luciferase activity was measured 24 h after transfection, and Renilla luciferase activity was used to normalize for transfection efficiency. Data are shown as percentage of control samples transfected with wild-type plasmid. The values represent means standard error of the mean (n = 6). * P< 0.05 by analysis of variance (ANOVA) with the post-hoc Newmann-Keuls test when comparing with control.

Next we analysed evolutionary conservation of 100 vertebrate species within the region of -583/-480. Three highly conserved regions were identified and they are -583/-570 (chr11:117187094-117187081, CGGCAGAGGGCATCC), -551/-544 (chr11:117187062-117187055, CCGGAAGC) and -528/-509 (chr11:117187039-117187020, GGGAAGACTACACTTCCCAG) (Fig. 5a). Nucleotide base level alignment analysis of five species, human and baboon from primate subset, mouse and rat from Euarchontoglires subset and elephant from Afrotheria subset, revealed that sequence motifs of regions -583/-569, -551/-544 and -528/-509 are exactly same between five species, although the single nucleotide of -582 (chr11:117187093) and -577 (chr11:117187088) in region -583/-569 is not conserved from 100 vertebrate species (Fig. 5a). The region of -570/-550 is not highly conserved between five species, which suggests that the sequence of -570/-550 region may have little effect on *BACE1* transcription.

TCE1 is located immediate upstream of the core promoter. To investigate whether it is orientation-dependent and activates other gene promoter, we constructed reporter plasmids p3BU-1149/-400TCE1R and pGL3p-TCE1. The TCE1 sequence in p3BU-1149/-400 was replaced with the sequence of reverse direction from CGGCAGAGGGC to GCCCTCTGCCG to generate p3BU-1149/-400TCE1R, and the TCE1 sequence was inserted in front of SV40 promoter in plasmid pGL3-Promoter to generate pGL3p-TCE1 (Fig. 5b,d). The luciferase activity of p3BU-1149/-400TCE1R was less than half of the control's activity, suggesting that TCE1 is orientation-dependent (Fig. 5c). However, placing the TCE1 sequence in front of SV40 promoter had no effect on luciferase activity of enhancer-less reporter plasmid pGL3-Promoter and it suggests that the function of TCE1 is specific to *BACE1* core promoter (Fig. 5e).

The distance between two cis-acting elements is important for gene transcription in some cases. Since deletion of the region -570 to -550 had no effects on *BACE1* basal transcription, the effect of the space between TCE1 and core promoter on BACE1 transcription was investigated to evaluate whether deleting region -570 to -550 had an effects on synergistic action of TCE1 and core promoter. Region -570 to -550 reverse mutation plasmid p3BU-1149/-400M570-551R and deletion mutation plasmid p3BU-1149/-400M570-551D were constructed with p3BU-1149/-400 as template (Fig. 5f). The sequence CCCAGACCCCTCTCCAGCCC of -570/-551 was changed to CCCGACCTCTCCCCAGACCC in p3BU-1149/-400M570-551R or deleted in p3BU-1149/-400M570-551D. Luciferase assay revealed that reverse mutation of region -570/-550 in p3BU-1149/-400M570-551R had no effects on luciferase activity and deletion mutation of region -570/-550 in p3BU-1149/-400M570-551D resulted in significant decrease in promoter activity (Fig. 5g). Taken together, TCE1 is an orientation-and position-dependent *cis*-acting promoter proximal element and a specific integral part of the *BACE1* core promoter.

### *BACE1* core promoter does not contain typical core promoter elements

**Figure 6:**
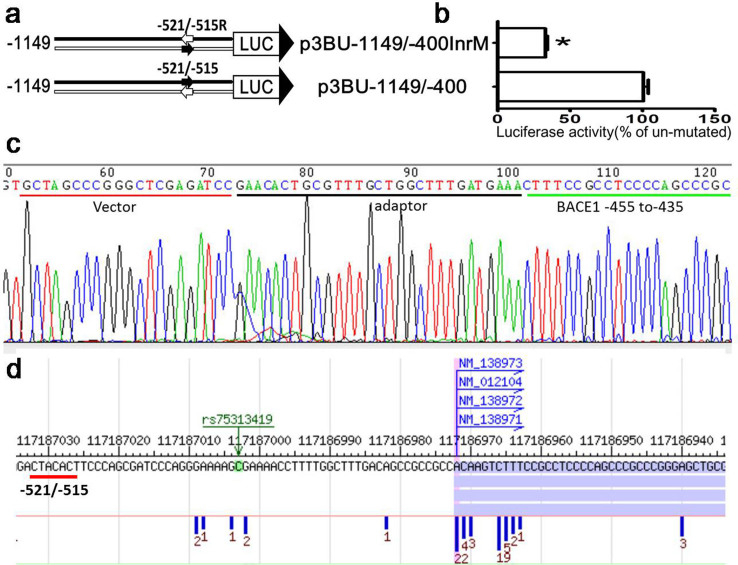
TCE2 is not an initiator of *BACE1* gene. (a) Schematic diagram of plasmid constructs. The two horizontal lines indicate two DNA strands of the *BACE1* promoter region; the box LUC with large arrowhead represents the coding sequence of the luciferase reporter gene and transcriptional direction; small arrow indicates tested DNA motif The numbers indicate the endpoints of each construct, with +1 as the adenine of the physiological translational start codon of the *BACE1* gene. (b) HEK293 cells were transiently transfected reporter plasmids. Firefly luciferase activity was measured 24 h after transfection, and Renilla luciferase activity was used to normalize for transfection efficiency. Data are shown as percentage of control samples transfected with wild-type plasmid. The values represent means standard error of the mean (n = 6). * P< 0.05 by analysis of t-test when comparing with control. (c) Sequencing result of the 5′ RACE product which was cloned into the pGL3-Basic vector. Sequence of the vector, adaptor and *BACE1* 5′UTR are shown underlined. The first nucleotide cytosine next to the 3′ of the adaptor is the TSS of human *BACE1* gene. (d) TSS distribution of *BACE1* gene in HEK293 cell from dbTSS(http://dbtss.hgc.jp/).The number under vertical blue bar represent TSS frequency identified from massively parallel sequencing of TSS.

The common core promoter elements include the Inr (initiator, yyanwyy), the TATA box (tatawaar), BRE (TFIIB recognition element, ssrcgcc), DPE (downstream core promoter element,rgwyvt), MTE (motif ten element,csarcssaacgs), and XCPE1(X core promoter element 1, dsgyggrasm)^42^. Sequence analysis reveals that *BACE1* gene core promoter region of -583/-480 has no TATA box, BRE, DPE,MTE, or XCPE1. However, there are two putative Inr elements. Inr (initiator) is a recognition site for the binding of transcriptional factor TFIID and has a consensus sequence of YYANWYY in human. Two putative Inr elements are located at -569/-563(CCAGACC) and -521/-515(CTACACT) respectively. Because upstream putative Inr site -569/-563 is located between-570 and -550 in which the sequence are not functional as shown before, and deleting region -570/-550 has no effects on *BACE1* basal transcription, upstream putative Inr is most likely not functional. Downstream putative Inr site is located at -521/-515 and is not consistent with published and reference gene TSS at GenBank, which are located between nucleotide 453 to 461 upstream of translational start site ATG. To verify whether it is functional Inr site, first we constructed plasmid p3BU-1149/-400InrM by replacing Inr site with an reverse Inr sequence (-521/-515 CTACACT was changed to tcACAtc) (Fig. 6a). The luciferase activity of reporter plasmid p3BU-1149/-400InrM was about 30% of the wildtype p3BU-1149/-400 (Fig. 6b), even though mutated sequence tcACAtc was still corresponding to Inr consensus sequence yyanwyy. Secondly we performed 5′-RACE experiment to define the actual TSS in reporter plasmid p3BU-1149/-400 and the result showed that the TSS is located at nucleotide -455 upstream of protein translational start site ATG and it is very close to known TSS of -461 in reference gene (NM_012104.4) (Fig. 6c). Because of technical limitation, 5′-RACE experiment can only define a few TSS each time. Next we applied dbTSS^43^ database to investigate the *BACE1* TSS, and the result revealed that *BACE1* TSS is not restricted into one nucleotide, but a region around -461. However, there is no tag count for the region -521/-515 (Fig. 6d). Taken together, downstream putative Inr site is not functional as an Inr. It suggests *BACE1* core promoter does not contain any known typical core promoter elements.

## Discussion

Phylogenetic analysis of DNA sequences is a useful strategy to identify DNA sequence motifs that have been strongly conserved through evolution and well-conserved sequence can provide much stronger evidence of functional relevance. A conservation analysis revealed 92% similarity between rat and mouse and 81% similarity between rat and human within the -600 bp upstream of the authentic ATG codon of *BACE1* gene, but similarity between rat and human in the region -1000 bp to -600 bp from ATG drops down to 39% and the similarity is lost beyond -1000 bp^21^. This observation indicates that the 600 bp region immediate upstream of ATG plays an important role in regulating *BACE1* expression, consistent with our results that deleting the upstream region of -583 has no effects on promoter activity. These results also suggest that human and mouse may have similar regulatory mechanism with 600 bp upstream of the ATG codon and have different regulatory mechanism beyond 600 bp region. Studies on promoter structure and transcriptional regulation of *BACE1* have been mainly focused on the rat and human genes. The *cis*-acting elements identified and verified experimentally in the rat and human *BACE1* gene need to be carefully considered in term of the location. The ones located over-600 bp from ATG, especially over-1000 bp region may play different roles in the transcriptional regulation of *BACE1* gene between rat and human. The *cis*-acting elements identified experimentally in human *BACE1* include NF-κB binding site^33^,^34^, STAT1 binding site^26^, hypoxia responsive element^22^, Sp1 binding site^19^, NFAT1-binding site^29^, and they are located at -3020/-2991, -2311/-2302, -1607/-1602, -1602/-1597, -954/-946 upstream of ATG, respectively. Those confirmed in rat *BACE1* include NF-κB binding site^35^, PPARγ responsive element^31^, YY1 binding site^28^, two GATA-1 binding site^21^, and they are located at -1521/-1511, -1356/-1338, -575/-560, 209/-200 and -106/-97 from ATG, respectively. Among these elements, only YY1 binding site and GATA-1 binding site are located 600 bp region immediate upstream of authentic ATG. Ge et al. reported a 91 nucleotides region as a minimal promoter at -223/-133 from ATG of human *BACE1* gene^44^. Two GATA-1 binding sites and the minimal promoter reported by Ge et al. are actually located at 5′ untranslated region (5′UTR) of mRNA, which have been proved to affect protein translation by a couple of groups^45^,^46^,^47^,^48^ and it may affect luciferase translation and activity if 5′UTR include in luciferase reporter plasmid. To focus on examining transcription regulation and to eliminate the interference from post-transcriptional regulation, when constructing reporter plasmids, we intentionally excluded the region of -400 bp to -1. The binding sequence of YY1 in rat *BACE1* promoter is located at -575/-560(ACTCCATCTCGGCAG) upstream of rat authentic ATG, same as the human sequence at -592/-578 (ACTCCACCTCGGCAG) except for one nucleotide difference at -586. Although putative YY1 in human gene overlap partially with TCE1, TCE1 is not like to be YY1 binding site based on following three reasons. First, only six nucleotides out of 15 in the 3′end of human putative YY1 binding site overlap with 5′end of -583 bp/-578 bp in TCE1. Secondly, deleting 9 nucleotides out of 15 at 5′ end in p3BU-583/-400 has similar luciferase activity as p3BU-700/-400, which include full YY1 binding sequence. It suggests that 5′ end 9 nucleotides of human putative YY1 binding site are not functional. Thirdly, according to reported YYI consensus sequence CGCCATnTT^49^, core motif of YY1 in rat corresponds to -589/-584 in human and this region does not overlap with TCE1. Therefore, TCE1 is a novel DNA motif to activate transcription of human *BACE1* gene.

RNAPII-transcribed genes typically contain two distinct families of *cis*-acting transcriptional control sequences, promoters and distal regulatory elements^50^. The promoter is the DNA sequence responsible for transcription initiation and can be divided into two sub-regions of a core promoter and a proximal promoter located immediate upstream of core promoter. Distal regulatory elements include enhancers, silencers, insulators, or locus control regions (LCR).The core promoter is generally defined as the minimal piece of DNA sequence sufficient to guide the assembly of transcription initiation complex at DNA chain and initiate a basal transcription^42^. Typical core promoter spans from around nucleotide -40 to +40 relative to transcription start site and consists of functional DNA motifs termed as core promoter elements. The core promoter elements are short DNA sequence in core promoter and confer distinct properties to the promoter. Our current knowledge of core promoter elements are primarily come from the analysis of focused core promoter in RNAPII-transcribed genes. Although parallel sequencing have been successfully used to study and identified TSS, enhancer and promoter in genome wide^39^,^51^,^52^, it is still challenging to define core promoter elements because of its considerable diversity and relatively few study on them, especially in those TATA-less promoters. By in-depth dissection of human *BACE1* promoter, our results demonstrated that *BACE1* gene core promoter lacks typical known core promoter elements including Inr, TATA box, BRE, DPE, MTE, and XCPE1. The newly identified TCE2 from our study is most likely to be a novel unknown core promoter element, which plays an important role in initiating a basal transcription and may be involved in different transcriptional mechanism.

In this study, we defined the boundary of human *BACE1* gene minimal and core promoter and found two important DNA motifs, TCE1 and TCE2. TCE1 is likely to be a promoter proximal element and TCE2 is a core promoter element. TCE1 and TCE2 function in a synergistic way and these two novel DNA motifs are essential for transcription of human *BACE1* gene. Future study on how these two motifs being affected by different stimuli to regulate BACE1 processing of APP could provide insights into the molecular mechanisms underlying Alzheimer pathogenesis and pharmaceutical potentials of targeting these motifs for AD treatment.

## Methods

### Cell culture and transfection

Human embryonic kidney 293 (HEK293) cells were cultured in Dulbecco's modified Eagle's medium supplemented with 10% fetal bovine serum, 1 mmol/L of sodium pyruvate, 2 mmol/L of L-glutamine and 1% penicillin-streptomycin at 37°C in a humidified incubator containing 5% CO2. Cells were grown to approximately 70 to 80% confluence and plasmid DNA were transfected using Lipofectamine 2000 reagent (Invitrogen) according to the supplier's protocol., 3.2 μg of total plasmid DNA were transfected into the cells cultured on a 35-mm-diameter dish for 5′-RACE and RNA extraction and 200 ng of total plasmid DNA per well were transfected into the cells cultured on a 48-well plate for luciferase reporter assay.

### Plasmid construction

*BACE1* promoter firefly luciferase reporter plasmid p3BU-1273/-400 contains human genomic sequence from –1273 to -400 relative to *BACE1* gene translating start site as +1. *BACE1* genomic fragment B1U-1273/-400 was amplified with primer B1U1273F (CCGCTCGAGAACCATACCGGCTTTTCTCCT)/B1U400R (CACAAGCTTCCACCATAATCCAGCTCG) and BAC DNA(RP11-970G21) as template. PCR product of B1U-1273/-400 was inserted in front of the firefly luciferase reporter gene in the promoterless reporter plasmid, pGL3-Basic (Promega) at XhoI/HindIII sites and inserted sequence was confirmed by DNA sequencing. The methods for constructing other plasmids are described in Supplementary Methods.

### Rapid amplification of 5′ complementary DNA ends (5′-RACE)

The plasmid p3BU-1149/-400 was transfected into the cells and total RNA was extracted from cells with TRI reagent following the manufacturer's protocol (Sigma). The 5′-RACE was carried out according to the FirstChoice RLM-RACE Instruction Manual (Ambion, Foster, CA, USA). Briefly, 10 μg RNA was treated with Calf Intestine Alkaline Phosphatase (CIP) and Tobacco Acid Pyrophosphatase (TAP), resulted RNA was ligated with 5′RACE Adapter (5′-GCUGAUGGCGAUGAAUGAACACUGCGUUUGCUGGCUUUGAUGAAA). The RNA with adapter was reverse transcripted into cDNA with SuperScript III First-Strand Synthesis System (Invitrogen) and amplified with outerprimer (5′outer primer: 5′-GCTGATGGCGATGAATGAACACTG/3′specific outer primer: 5′-TTCCAGGAACCAGGGCGTATCTCT). 3′ specific outer reverse primer matches luciferase coding region and start from minus strand +115, which is relative to luciferase gene translating start site as+1. Above PCR product was re-amplified with inner primer (5′inner primer: 5′-CGCGGATCCGAACACTGCGTTTGCTGGCTTTGATG/3′specific inner primer:5′-GTGAAGCTTGGTTCCATCTTCCAGCGGATAGAAT). 3′ specific inner reverse primer matches luciferase coding region and start from minus strand +66. Resulted PCR products were digested with BamHI/HindIII and cloned into plasmid pGL3-Basic at Bgl II/HindIII sites. Positive clone was sequenced with pGL3-Basic sequencing primer 5′-CTAGCAAAATAGGCTGTCCC to determine the transcription initiation site of *BACE1* in p3BU-1149/-400.

### Luciferase assay

Cells cultured in 48-well plates were co-transfected with 180 ng of BACE1-luciferase reporter plasmids and 20 ng of p3PRluc plasmid per well. P3PRluc has *Renilla* luciferase gene and serves as transfection control to normalize for the transfection efficiency of various luciferase reporter constructs. Twenty-four hours after transfection, cells were harvested and luciferase activities were measured using the dual-luciferase reporter assay system according to the supplier's protocol (Promega). The firefly luciferase activity was normalized to the Renilla luciferase activity and expressed as relative luciferase units (RLUs) to reflect the promoter activity.

## Author Contributions

W.Z. designed the research, performed experiments and wrote the manuscript; W.S. planned the research and wrote the manuscript; Y.X. and S.M. performed experiments; J.W., S.L. and J.L. constructed plasmids; H.L. constructed plasmids and analyzed data; T.L. discussed the project and provided reagents.

## Supplementary Material

Supplementary Information Supplementary Methods
