# Supplementary material for: Two novel DNA motifs are essential for BACE1 gene transcription
Source: Sci Rep. 2014 Oct 31;4:6864. doi: 10.1038/srep06864 (PMC4215305; doi:10.1038/srep06864)
Supplement: Supplementary Information — Supplementary Methods [file srep06864-s1.pdf]

Two novel DNA motifs are essential for *BACE1* gene transcription

Yan Xiang, Shasha Meng, Jinfeng Wang, Songyang Li, JingruLiu, Hongmei Li, Tingyu Li, Weihong Song and Weihui Zhou

SupplementaryMethods

Plasmid construction

Plasmids p3BU-1149/-400, p3BU-919/-400, p3BU-800/-400, p3BU-700/-400, p3BU-583/-400, p3BU-574/-400, p3BU-560/-400, p3BU-540/-400, p3BU-520/-400, p3BU-500/-400,p3BU-583/400M580T, p3BU-583/400M578A , p3BU-583/400M580T578A, p3BU-583/-420, p3BU-583/-440, p3BU-583/-460, p3BU-583/-480 were constructed by amplifying DNA fragments with corresponding primer pairs and DNA of p3BU-1273/-400 as template, resulted PCR products were inserted into pGL3-Basic at XhoI/HindIII sites. To construct plasmid p3BU-583/-500, p3BU-583/-510, p3BU-570/-480 p3BU-560/-480，p3BU-550/-480，p3BU-540/-480，p3BU-530/-480，p3BU-520/-480，p3BU-510/-480, DNA fragments were amplified with corresponding 5’ phosphorylated primer pairs and DNA of p3BU-583/-480 as template, resulted PCR products were self-ligated to generate circle plasmids. To construct plasmid p3BU-583/-400M510C, p3BU-583/-400M482A and p3BU-583/-400M510C482A, DNA fragments were amplified with corresponding 5’ phosphorylated primer pairs and DNA of p3BU-583/-400 as template, resulted PCR products were self-ligated to generate circle plasmids. To construct plasmid p3BU-583/-400M578A510C, p3BU-583/-400M578A482A, DNA fragments were amplified with corresponding 5’ phosphorylated primer pairs and p3BU-583/-400M510C or p3BU-583/-400M482A as

templates, resulted PCR products were self-ligated to generate circle plasmids. To construct plasmid p3BU-1149/-400M578A, p3BU-1149/-400M510C , p3BU-1149/-400M482A, p3BU-1149/-400M578x, p3BU-1149/-400M578y, p3BU-1149/-400M578z, p3BU-1149/-400M510x, p3BU-1149/-400M510y, p3BU-1149/-400M510z, p3BU-1149/-400M550-551R, p3BU-1149/-400M550-551D,p3BU-1149/-400MIZ，p3BU-1149/-400TCE1R, p3BU-1149/-400InrM, DNA fragments were amplified with corresponding 5' phosphorylated primer pairs and DNA of p3BU-1149/-400 as template, resulted PCR products were self-ligated to generate circle plasmids.To construct plasmid p3BU-1149/-400M578A510C, p3BU-1149/-400M578A482A, DNA fragments were amplified with corresponding 5' phosphorylated primer pairs and p3BU-1149/-400M510C or p3BU-1149/-400M482A as templates and, resulted PCR products were self-ligated to generate circle plasmids. To construct plasmid pGL3basicEGFP, pGL3promoter-EGFP, p3BU-1149/400EGFP and p3BU-1149/400 M578A510CEGFP, EGFP DNA fragment was amplified with primers CAGAAGCTTCGCCACCATGGTGAGCAAGG/CAGTCTAGATTACTTGTACAG CTCGTC and plasmid pEGFP-N2 as template, PCR product was inserted into plasmid pGL3-Basic, pGL3-Promoter, p3BU-1149/400 and p3BU-1149/400 M578A510C at HindII/XbaI sites to replace luciferase gene . For construction of p3BU-584/-480R, DNA fragment of BACE1U-584/-480 was amplified with primer B1U-584fHind / B1U-480rXho and plasmid p3BU1149-400B as template. PCR product of B1U-584/-480 was inserted into pGL3basic at HindIII/XhoI . To construct plasmid pGL3p-TCE1, a pair of primers B1U583XhF/B1U573XhR that complementary with each other was annealed and inserted in front of SV40 promoter in pGL3-Promoter vector at XhoI site. For construction of other single nucleotide

substitution plasmids, DNA fragments were amplified with DNA of p3BU-1149/-400 as template and corresponding 5’phosphorylated primer pairs, resulted PCR products were self-ligated to generate circle plasmids. All plasmids were confirmed by DNA sequencing. Corresponding primers were listed in following Table.

Plasmid name	Forward primer	Reverse primer
p3BU-1149/-400	B1U1149F:CCGCTCGAGCCATTTCTCCTCAGTC	B1U400R:CACAAGCTTCCACCATAATCCAGCTCG
p3BU-919/-400	B1U919F:CCGCTCGAGCAATCTCTGCTCGTGAA	B1U400R
p3BU-800/-400	B1U800F:CCGCTCGAGACATCGTATAGTGTGAAAC	B1U400R
p3BU-700/-400	B1U700F:CCGCTCGAGCCTGGCATACAGTGGGTG	B1U400R
p3BU-583/-400	B1U583F:CCGCTCGAGCGGCAGAGGGCATCCCAGAC	B1U400R
p3BU-574/-400	B1U574F:CCGCTCGAGGCATCCCAGACCCCTCTC	B1U400R
p3BU-560/-400	B1U560F:CCGCTCGAGCCTCTCCAGCCCCGGAAG	B1U400R
p3BU-540/-400	B1U540F:CCGCTCGAGATTGCTGCCATGGGAAG	B1U400R
p3BU-520/-400	B1U520F:CCGCTCGAGTACACTTCCCAGCGATCC	B1U400R
p3BU-500/-400	B1U500F:CCGCTCGAGGGAAAAGCGAAAACCTT	B1U400R
p3BU-583/-400M580T	B1U583T580F:CCGCTCGAGCGGTAGAGGGCATCCCAGAC	B1U400R
p3BU-583/-400M578A	B1U583A578F:CCGCTCGAGCGGCAAAGGGCATCCCAGAC	B1U400R
p3BU-583/-400M578A580T	B1U583T580A578F:CCGCTCGAGCGGTAAAGGGCATCCCAGAC	B1U400R
p3BU-583/-420	B1U583F	B1U420R:CACAAGCTTGCTCGCAGCTCCCGGGCG
p3BU-583/-440	B1U583F	B1U440R:CACAAGCTTCTGGGGAGGCGGAAAGAC
p3BU-583/-460	B1U583F	B1U460R:CACAAGCTTGTGGCGGCGGCTGTCAAAG
p3BU-583/-480	B1U583F	B1U480R:CACAAGCTTCAAAAGGTTTTCGCTTTTC

p3BU-583/-500	p3BasicPF:AAGCTTGGCATTCCGGTAC	B1U500PR:CTGGGATCGCTGGGAAGTG;
p3BU-583/-510	p3BasicPF	B1U510PR: TGGGAAGTGTAGTCTTCC
p3BU-570/-480	B1U570PF：CCCAGACCCCTCTCCAGCC	p3BasicPR：CTCGAGCCCGGGCTAGCAC
p3BU-560/-480	B1U560PF:TCTCCAGCCCCGGAAGCCG	p3BasicPR
p3BU-550/-480	B1U-550PF: CGGAAGCCGGATTGCCTGC	p3BasicPR
p3BU-540/-480	B1U-540PF: ATTGCCTGCCATGGGAAGAC	p3BasicPR
p3BU-530/-480	B1U530PF:ATGGGAAGACTACACTTCC	p3BasicPR
p3BU-520/-480	B1U520PF:TACACTTCCCAGCGATCC	p3BasicPR
p3BU-510/-480	B1U510PF:AGCGATCCCAGGGAAAAGC	p3BasicPR
p3BU-583/-400M510C	B1U482TF:AAAGCGAAAACCTTTTGGCTTTGACAGC	B1U510CMR:TCCCTGGGATCGCGGGGAAGTGTAGTCT
p3BU-583/-400M482A	B1U482AMF:AAAGCGAAAACCTTAT GGCTTTGACAGC	B1U510AR:TCCCTGGGATCGCTGGGAAGTGTAGTCT
p3BU-583/-400M510C482A	B1U482AMF	B1U510CMR
p3BU-583/-400M578A510C	B1U583CAf:CGGCAAAGGGCATCCCAGAC	p3BasicPR:
p3BU-583/-400M578A482A	B1U583CAf	p3BasicPR
p3BU-1149/-400M578A	B1U583CAf	B1U584R:AGGTGGAGTCCGCCCTGC
p3BU-1149/-400M510C	B1U482TF	B1U510CMR
p3BU-1149/-400M482A	B1U482AMF	B1U510AR
p3BU-1149/-400M578x	B1U583PfMx:CAACAGAGGGCATCCCAGAC	B1U584R
p3BU-1149/-400M578y	B1U583PfMy:CGGAGGAGGGCATCCCAGAC	B1U584R
p3BU-1149/-400M578z	B1U583PfMz:CGGCAGTAGGCATCCCAGAC	B1U584R
p3BU-1149/-400M510x	B1U482TF	B1U510PrMx:TCCCTGGGATCGCTGGTTAGTGTAGTCT
p3BU-1149/-400M	B1U482TF	B1U510PrMy:TCCCTGGGAT

510y		CGCTTTGAAGTGTAGTCT
p3BU-1149/-400M 510z	B1U482TF	B1U510PrMz:TCCCTGGGAT CTTTGGGAAGTGTAGTCT
p3BU-1149/-400M 578A510C	B1U583CAf	B1U584R
p3BU-1149/-400M 578A482A	B1U583CAf:	B1U584R
p3BU-1149/-400M 550-551R	B1UR570-535PF:CCCCAGAC CCCGGAAGCCGGATTGCC	B1UR551-586PR:AGAGGTCG GGATGCCCTCTGCCGAGG
p3BU-1149/-400M 550-551D	B1U550PF:CGGAAGCCGGATT GCCTGC	B1U571PR:ATGCCCTCTGCC GAGGTGG
p3BU-584/-480Br	B1U-584fHind:CACAAGCTTCG GCAGAGGGCATCCCAGAC	B1U-480rXho:CCGCTCGAGC AAAAGGTTT TCGCTTTTC
p3BU-1149/-400M IZ	MIZ F:CCTCTGCC AGACCCCTCTCCAGC	MIZ R:GCATCCCG AGGTGGAGTCCGCC
p3BU-1149/-400T CE1R	TCE1F:CTGCCATCC CAGACCCCTCTCCAG	TCE1R:AGGGCAGGT GGAGTCCGCCCTGC AAG
p3BU-1149/-400In rM	InrMF:TCACATCTCCCAGCGA TCCCAG	InrMR:TCTTCCCATGGCAGG CAATC
pGL3p-TCE1	B1U583XhF:TCGAGCGGCAGA GGGCC,	B1U573XhR:TCGAGGCCCTC TGCCGC
p3BU-1149/-400M 584A	B1U-F583 CGGCAGAGGG CATCCCAGAC	B1U-R584T/A: TGGTGGAGTC CGCCCTGC
p3BU-1149/-400M 584C	B1U-F583	B1U-R584T/C: GGGTGGAGTC CGCCCTGC
p3BU-1149/-400M 584G	B1U-F583	B1U-R584T/G: CGGTGGAGTC CGCCCTGC
p3BU-1149/-400M 583A	B1U-F583C/A:AGGCAG AGGGCATCCCAGAC	B1U-R584:AGGTGG AGTCCGCCCTGC
p3BU-1149/-400M 583T	B1U-F583C/T:TGGCAG AGGGCATCCCAGAC	B1U-R584
p3BU-1149/-400M 583G	B1U-F583C/G:GGGCAGAGGG CATCCCAGAC	B1U-R584
p3BU-1149/-400M 582A	B1U-F582G/A:CAGCAGAGGG CATCCCAGAC	B1U-R584
p3BU-1149/-400M 582T	B1U-F582G/T:CTGCAGAGGGC ATCCCAGAC	B1U-R584
p3BU-1149/-400M 582C	B1U-F582G/C:CCGCAGAGGG CATCCCAGAC	B1U-R584
p3BU-1149/-400M 581A	B1U-F581G/A:CGACAGAGGG CATCCCAGAC	B1U-R584
p3BU-1149/-400M 581C	B1U-F581G/C:CGCCAGAGGG CATCCCAGAC	B1U-R584

p3BU-1149/-400M 581T	B1U-F581G/T:CGTCAGAGGG CATCCCAGAC	B1U-R584
p3BU-1149/-400M 580A	B1U-F580C/A:CGGAAGAGGG CATCCCAGAC	B1U-R584
p3BU-1149/-400M 580T	B1U-F580C/T:CGGTAGAGGGC ATCCCAGAC	B1U-R584
p3BU-1149/-400M 580G	B1U-F580C/G:CGGGAGAGGG CATCCCAGAC	B1U-R584
p3BU-1149/-400M 579C	B1U-F579A/C:CGGCCGAGGG CATCCCAGAC	B1U-R584
p3BU-1149/-400M 579T	B1U-F579A/T:CGGCTG AGGGCATCCCAGAC	B1U-R584
p3BU-1149/-400M 579G	B1U-F579A/G:CGGCGG AGGGCATCCCAGAC	B1U-R584
p3BU-1149/-400M 578C	B1U-F578G/C:CGGCAC AGGGCATCCCAGAC	B1U-R584
p3BU-1149/-400M 578T	B1U-F578G/T:CGGCAT AGGGCATCCCAGAC	B1U-R584
p3BU-1149/-400M 577C	B1U-F577A/C:CGGCAGCGGG CATCCCAGAC	B1U-R584
p3BU-1149/-400M 577T	B1U-F577A/T:CGGCAGTGGGC ATCCCAGAC	B1U-R584
p3BU-1149/-400M 577G	B1U-F577A/G:CGGCAGGGGG CATCCCAGAC	B1U-R584
p3BU-1149/-400M 576A	B1U-F576G/A:CGGCAG AAGGCATCCCAGAC	B1U-R584
p3BU-1149/-400M 576T	B1U-F576G/T:CGGCAGATGGC ATCCCAGAC	B1U-R584
p3BU-1149/-400M 576C	B1U-F576G/C:CGGCAGACGG CATCCCAGAC	B1U-R584
p3BU-1149/-400M 575A	B1U-F575G/A:CGGCAG AGAGCATCCCAGAC	B1U-R584
p3BU-1149/-400M 575T	B1U-F575G/T:CGGCAG AGTGCATCCCAGAC	B1U-R584
p3BU-1149/-400M 575C	B1U-F575G/C:CGGCAG AGCGCATCCCAGAC	B1U-R584
p3BU-1149/-400M 574T	B1U-F574G/T:TCATCCCAGAC CCCTCTCCAGC	B1U-R575:CCTCTG CCGAGGTGGAGTCC
p3BU-1149/-400M 574C	B1U-F574G/C:CCATCCCAGAC CCCTCTCCAGC	B1U-R575
p3BU-1149/-400M 574A	B1U-F574G/A:ACATCCCAGAC CCCTCTCCAGC	B1U-R575
p3BU-1149/-400M	B1U-F573C/T:GTATCCCAGAC	B1U-R575

573T	CCCTCTCCAGC	
p3BU-1149/-400M 573G	B1U-F573C/G:GGATCCCAGAC CCCTCTCCAGC	B1U-R575
p3BU-1149/-400M 573A	B1U-F573C/A:GAATCCCAGAC CCCTCTCCAGC	B1U-R575
p3BU-1149/-400M 572T	B1U-F572A/T:GCTTCCCAGAC CCCTCTCCAGC	B1U-R575
p3BU-1149/-400M 572C	B1U-F572A/C:GCCTCCCAGAC CCCTCTCCAGC	B1U-R575
p3BU-1149/-400M 572G	B1U-F572A/G:GCGTCCCAGAC CCCTCTCCAGC	B1U-R575
p3BU-1149/-400M 516A	B1U-F516C/A:TACAA TTCCCAGCGATCCCAGG	B1U-R521:GTCTTCCCATGG CAGGCAATC
p3BU-1149/-400M 516T	B1U-F516C/T:TACATTTCCCA GCGATCCCAGG	B1U-R521
p3BU-1149/-400M 516G	B1U-F516C/G:TACAGTTCCCA GCGATCCCAGG	B1U-R521
p3BU-1149/-400M 515A	B1U-F515T/A:TACACA TCCCAGCGATCCCAGG	B1U-R521
p3BU-1149/-400M 515C	B1U-F515T/C:TACACC TCCCAGCGATCCCAGG	B1U-R521
p3BU-1149/-400M 515G	B1U-F515T/G:TACACG TCCCAGCGATCCCAGG	B1U-R521
p3BU-1149/-400M 514A	B1U-F514T/A:ACCCAGC GATCCCAGGGAAAAGC	B1U-R515:AGTGTAG TCTTCCCATGGCAGGC
p3BU-1149/-400M 514C	B1U-F514 T/C:CCCCAGC GATCCCAGGGAAAAGC	B1U-R515
p3BU-1149/-400M 514G	B1U-F514 T/G:GCCCAGC GATCCCAGGGAAAAGC	B1U-R515
p3BU-1149/-400M 513A	B1U-F513C/A:TACCAGC GATCCCAGGGAAAAGC	B1U-R515
p3BU-1149/-400M 513T	B1U-F513C/T:TTCCAGC GATCCCAGGGAAAAGC	B1U-R515
p3BU-1149/-400M 513G	B1U-F513C/G:TGCCAGC GATCCCAGGGAAAAGC	B1U-R515
p3BU-1149/-400M 512G	B1U-F512C/G:TCGCAGC GATCCCAGGGAAAAGC	B1U-R515
p3BU-1149/-400M 512A	B1U-F512C/A:TCACAGC GATCCCAGGGAAAAGC	B1U-R515
p3BU-1149/-400M 512T	B1U-F512C/T:TCTCAGC GATCCCAGGGAAAAGC	B1U-R515
p3BU-1149/-400M 511A	B1U-F511C/A:TCCAAGC GATCCCAGGGAAAAGC	B1U-R515

p3BU-1149/-400M 511T	B1U-F511C/T:TCCTAGC GATCCCAGGGAAAAGC	B1U-R515
p3BU-1149/-400M 511G	B1U-F511C/G:TCCGAGC GATCCCAGGGAAAAGC	B1U-R515
p3BU-1149/-400M 510G	B1U-F510A/G:TCCCGGC GATCCCAGGGAAAAGC	B1U-R515
p3BU-1149/-400M 510T	B1U-F510A/T:TCCCTGC GATCCCAGGGAAAAGC	B1U-R515
p3BU-1149/-400M 509C	B1U-F509G/C:CCCACC GATCCCAGGGAAAAGC	B1U-R515
p3BU-1149/-400M 509A	B1U-F509G/A:CCCAAC GATCCCAGGGAAAAGC	B1U-R515
p3BU-1149/-400M 509T	B1U-F509G/T:TCCCATC GATCCCAGGGAAAAGC	B1U-R515
p3BU-1149/-400M 508A	B1U-F508C/A:CCCAGA GATCCCAGGGAAAAGC	B1U-R515
p3BU-1149/-400M 508T	B1U-F508C/T:CCCAGT GATCCCAGGGAAAAGC	B1U-R515
p3BU-1149/-400M 508G	B1U-F508C/G:CCCAGG GATCCCAGGGAAAAGC	B1U-R515
p3BU-1149/-400M 507A	B1U-F507G/A:AATCCCAGGG AAAAGCGAA	B1U-R508:CTGGGA AGTGTAGTCTT
p3BU-1149/-400M 507T	B1U-F507G/T:TATCCCAGGGA AAAGCGAA	B1U-R508
p3BU-1149/-400M 507C	B1U-F507G/C:CATCCCAGGGA AAAGCGAA	B1U-R508
p3BU-1149/-400M 506T	B1U-F506A/T: GTTCCCAGGGAAAAGCGAA	B1U-R508
p3BU-1149/-400M 506C	B1U-F506A/C: GCTCCCAGGGAAAAGCGAA	B1U-R508
p3BU-1149/-400M 506G	B1U-F506A/G: GGTCCCAGGGAAAAGCGAA	B1U-R508
p3BU-1149/-400M 505A	B1U-F505T/A: GAACCCAGGGAAAAGCGAA	B1U-R508
p3BU-1149/-400M 505C	B1U-F505T/C: GACCCCAGGGAAAAGCGAA	B1U-R508
p3BU-1149/-400M 505G	B1U-F505T/G: GAGCCCAGGGAAAAGCGAA	B1U-R508
